# Interaction between adipocytes and macrophages participates in chick subcutaneous adipose tissue angiogenesis under cold stress conditions

**DOI:** 10.1080/10495398.2024.2400212

**Published:** 2024-09-17

**Authors:** Yuelang Zhang, Jingxuan Li, Shanhe Wang, Mingli Wu, Haidong Zhao

**Affiliations:** a Hainan Institute of Zhejiang University, Sanya, China; b College of Animal Science and Technology, Yangzhou University, Yangzhou, China; c College of Animal Science and Technology, Northwest A&F University, Yangling, Shaanxi, China; d Guangxi Key Laboratory of Brain and Cognitive Neuroscience, Guilin Medical University, Guilin, China; e Scientific Research Center, Guilin Medical University, Guilin, China

**Keywords:** Cold stress, chick, macrophage, adipocyte, angiogenesis

## Abstract

Previous studies have shown that subcutaneous adipose tissue is an important energy supply organ for chicks before and after birth, except yolk. So far, the significance of large deposits of subcutaneous adipose tissue in chicks is unclear. Therefore, this study takes the information interaction between adipocytes and macrophages as the starting point to explore whether adipocytes and macrophages could participate in adipose tissue fibrosis, angiogenesis, adaptive thermogenesis and other related functions in a specific metabolic environment. Under cold stress, the expression levels of genes related to lipidolysis, lipid transport and fatty acid oxidation in adipose tissue of chicks were significantly increased, but the expression levels of genes related to mitochondrial uncoupling were not significantly changed. Through Masson staining of adipose tissue of chicks under cold stress, it was found that the level of vascularization in adipose tissue of chicks was significantly increased. We found that the interaction between adipocyte and macrophage could participate in the angiogenesis related process of adipocytes in chicks through the HIF1A–VEGFA pathway. The analysis of lipid metabolism in subcutaneous adipose tissue of chicks from the perspective of cell heterogeneity will expand the understanding of lipid metabolism in chicks and provide a theoretical basis for chick rearing.

## Introduction

Our research team has provided a comprehensive description of the developmental process of subcutaneous adipose tissue in chicken embryos and chicks. At 12 days of embryonic stage, subcutaneous adipose tissue initiates its formation in the neck, chest and legs. By the time of hatching, it continues to expand and constitutes approximately 2% of body weight.^1^ However, there is limited accumulation of abdominal fat at this stage. Therefore, it is speculated that significant deposition of subcutaneous adipose tissue plays a crucial role for chick development. During the chick stage, unlike other organs, subcutaneous adipose tissue undergoes dynamic changes which are believed to be associated with environmental adaptability.^1^ The distribution pattern of subcutaneous fat in chickens resembles that observed in brown fat in rodents and is predominantly found in areas with sparse feathers.^1^^,^^2^ It could be inferred that subcutaneous adipose tissue may contribute to thermoregulation and non-shivering thermogenesis (NST) processes.^3^^,^^4^ Through extensive heterogeneity analysis on chick subcutaneous adipocytes conducted by our research team, a substantial number of recruited macrophages have been identified within them. Transcriptomic studies along with related cellular biology investigations have revealed potential interactions between adipocytes and macrophages involved in fatty acid transport processes within chick’s subcutaneous adipose tissue.^5^ However, whether these interactions also play a role in fatty acid oxidation under cold stress conditions or NST functions remains unknown.

The thermogenic effect of the organism is a temperature regulation mechanism that warm-blooded animals must possess in response to environmental temperatures lower than their thermal neutral zone. The thermogenesis could be primarily divided into two types: obligatory thermogenesis and adaptive thermogenesis.^6^ Adaptive thermogenesis could be categorized into shivering thermogenesis (ST) and NST, with the former predominantly occurring in muscles and the latter referring to the uncoupling protein (UCP)-mediated mitochondrial uncoupling effect regulated by thyroid hormones, particularly in brown adipose tissue.^7^ Currently, there is still controversy surrounding whether chickens possess brown adipose tissue.^8^ However, due to the absence of *UCP1* gene in chickens, their ability for NST function in adipose tissues faces significant obstacles. Only *UCP3* gene has been identified in chickens, with most studies focusing on muscle tissues and only a small amount expressed in adipose tissues.^9^^,^^10^ Whether it can be activated by cold stress signals in chick remains unreported. In addition to NST, lipids stored in subcutaneous adipose tissue can undergo lipolysis to generate free fatty acids, which are then transported to relevant tissues of the body, particularly muscle tissue.^5^ The presence of *UCP3* in muscle tissue facilitates the uncoupling process that allows fatty acids to produce the necessary heat for the body.^11^ The transport of lipid acids is dependent on the level of vascularization in adipose tissue. However, there have been no reports on whether the interaction between adipocytes and macrophages is involved in the physiological process of angiogenesis in subcutaneous adipose tissue of chicks.

The objective of this study is to establish a chick cold stress animal model for evaluating the morphological characteristics of subcutaneous adipose tissue in chicks, as well as assessing fatty acid metabolism, immune cell recruitment, thermogenic function, tissue fibrosis and angiogenesis. Furthermore, by incorporating a model that examines the interaction between adipocytes and macrophages, we aim to investigate the role of subcutaneous adipose tissue in thermogenic function in chicks. This research provides novel insights into the functionality of avian adipose tissue, particularly subcutaneous adipose tissue in chicks, and offers valuable scientific evidence for controlling environmental temperature during poultry brooding.

## Materials and methods

All experimental procedures were performed in accordance with the Regulations for the Administration of Affairs Concerning Experimental Animals approved by the State Council of the People’s Republic of China. The study was approved by the Institutional Animal Care and Use Committee of Northwest A&F University (Permit Number: NWAFAC1019).

### Animals

Lohmann pink chicken embryos and chicks used in the current study were bought from the Yangling Julong Poultry Industry Co. Ltd. (Yangling, China). Incubation conditions: E (Embryonic) 1–E19, 37.8 °C, E19–E21, 37–37.5 °C (Qingdao Xinyi Electronic Equipment Co., Ltd. Qingdao, China). After hatching, chicks were allowed ad libitum access to water and feed. Twenty chicks were divided into two groups: cold stress group (4 °C) and NC group (30 °C). Chicks were humanely euthanized by cervical dislocation after 36 h and subcutaneous adipose tissues were collected. Samples for RNA and protein detection were stored at −80 °C and the other samples for paraffin section were dipped in 4% formaldehyde until analysis. Besides, the heart, liver, spleen, lung, kidney, intestine, pancreas, gizzard and adipose tissue were collected from different development stages, including E14, E20, D1 and D9.

### RNA isolation and quantitative real-time PCR

Quantitative real-time PCR (qPCR) was performed to verify the related gene expression pattern of two groups. Total RNA for qPCR was extracted using RNAiso plus kit (Takara, Tokyo, Japan). Total RNA concentration and quality were evaluated using Nanodrop 1000 (Thermo, Massachusetts, USA). Reverse transcription of RNA to cDNA (Takara, Tokyo, Japan) was performed before qPCR, carried out in the Y480 Real-Time PCR detection system (Roche, Basel, Switzerland) utilizing SYBR green detection (Takara, Tokyo, Japan). Primers designing used NCBI primer and their Tm were close to 60 °C (Table S1). The amplification protocol was as follows: 95 °C for 30 s, followed by 50 cycles of 95 °C 10 s and 60 °C for 30 s. Melt curve analysis was performed between 55 and 95 °C, with a 0.5 °C increment every 5 s. Samples were run in triplicate. All mRNA expression levels were normalized to the arithmetic mean of ACTB, the mRNA relative expression were quantified using the 2^−ΔΔCt^ method.

### Haematoxylin and eosin staining, toluidine blue staining, immunohistochemistry and Masson staining

Chick subcutaneous adipose tissue were fixed in 4% paraformaldehyde solution in phosphate buffer saline (PBS) for 2 days and processed through a series of procedures including dehydration, paraffin embedding, sectioning and staining. All these procedures were performed by Wuhan Servicebio Technology Co., Ltd (Wuhan, China), including haematoxylin and eosin (HE), toluidine blue and immunohistochemistry (IHC). Primary antibody information: LGALS3 (Galectin 3) (Servicebio, Wuhan, China).

### Cell culturing

Chicken adipocyte culturing: chick embryos were sacrificed by cervical dislocation and subcutaneous adipose tissue was placed in a 10 mL tube with 1 mL PBS at room temperature, cut into tiny bits with scissors. Adipocyte was obtained by collagenase II digestion (Sangong, Shanghai, China). The condition: 0.1 mg/mL collagenase II in 1× Hanks’ balanced salt solution, incubating at 37 °C for 1 h and shook every 5 min. Adipocyte was collected from cell suspension though 200 mesh cell strainer and maintained in DMEM high glucose containing 10% foetal bovine serum (FBS) (Solarbio Science & Technology Co., Ltd, Beijing, China), 5% chicken serum (Solarbio Science & Technology Co., Ltd, Beijing, China), 50 U/mL penicillin and 50 μg/mL streptomycin (Sangong, Shanghai, China).

Chicken macrophage culturing: chicken monocyte-derived macrophages were collected in chicken blood by density gradient separation (Solarbio Science & Technology Co., Ltd, Beijing, China) and maintained in RPMI1640 containing 10% FBS, 5% chicken serum, 50 U/mL penicillin and 50 μg/mL streptomycin.

RAW264.7 macrophage was maintained in RPMI1640 containing 10% FBS, 50 U/mL penicillin and 50 μg/mL streptomycin.

3T3-L1 adipocyte was maintained in DMEM high glucose containing 10% FBS, 50 U/mL penicillin and 50 μg/mL streptomycin.

To induce adipogenic differentiation of adipocyte, the basal medium was supplemented with 100 μmol/L sodium oleate (Sangong, Shanghai, China) dissolved in sterile deionized water. The differentiation medium was changed daily for a week.

To induce lipidolysis of adipocyte, the basal medium was supplemented with 20 µM forskolin (FSK) (Sangong, Shanghai, China) for 6 h.

Cell coculture was performed using transwell insert. Lipidolysis-induced 3T3-L1 adipocyte and macrophage were cocultured for 24 h, RNA and protein were collected and extracted for qPCR and western blot detecting.

### Western blot

Total protein of adipocyte was lysed with RIPA lysis buffer (1 mM MgCl_2_, 10 mM Tris-HCl pH 7.4, 1% Triton X-100, 0.1% sodium dodecyl sulphate (SDS) and 1% Nonidet P40 cocktail). The proteins were collected and quantified by using the BCA^™^ Protein Assay Kit (Sangon, Shanghai, China). The proteins were separated by 5–12% SDS-PAGE and transfected to polyvinylidene fluoride membranes and blocked in 5% nonfat milk for 1 h at room temperature. The membranes were incubated overnight with the following primary antibodies: ATGL (triacylglycerol lipase) (Sangon, Shanghai, China), FABP4 (fatty acid-binding protein 4) (Sangon, Shanghai, China), FABP5 (fatty acid-binding protein 5) (Sangon, Shanghai, China), CPT1A (carnitine palmitoyltransferase 1 A) (Sangon, Shanghai, China), CPT2 (carnitine palmitoyltransferase 2) (Sangon, Shanghai, China). Then, blots were washed three times with PBS and were incubated with horseradish peroxidase-conjugated goat anti-rabbit IgG (Sangon, Shanghai, China) for 1 h at room temperature. The blots were examined by using ECL reagents (Sangon, Shanghai, China) according to the manufacturer’s instructions. The intensity of the bands was quantified by using Image Lab^™^ Software (Bio-Rad).

### Thermal imaging of living body adipose tissue under the skin of chicks

Infrared thermal imaging was used to obtain whole-body thermal radiation images of chicks, allowing for the evaluation of temperature anomalies in their subcutaneous adipose tissue.

### Detection of FFA in chick serum

Free fatty acid (FFA) in the blood of chicks was detected using the detection kit. The detection procedure and result were calculated according to the kit instructions.

### RNA-seq data analysis

RNA-seq data presented in this study can be found in online repositories. The names of the repository/repositories and accession number(s) can be found below: https://www.ncbi.nlm.nih.gov/sra/PRJNA811769.

### Statistics

Statistical analysis was performed using SPSS software version 21.0 (SPSS Inc., Chicago, IL) or Microsoft Excel (Microsoft). Data analysis involved unpaired two-tailed Student’s *t* test for two groups and one-way ANOVA for more than two groups. Data shown are average ± standard error of the mean. The *P* value of <0.05 was considered to be statistically significant.

## Results

### Effect of cold stress on subcutaneous adipose tissue weight of chicks

**Figure 1.**
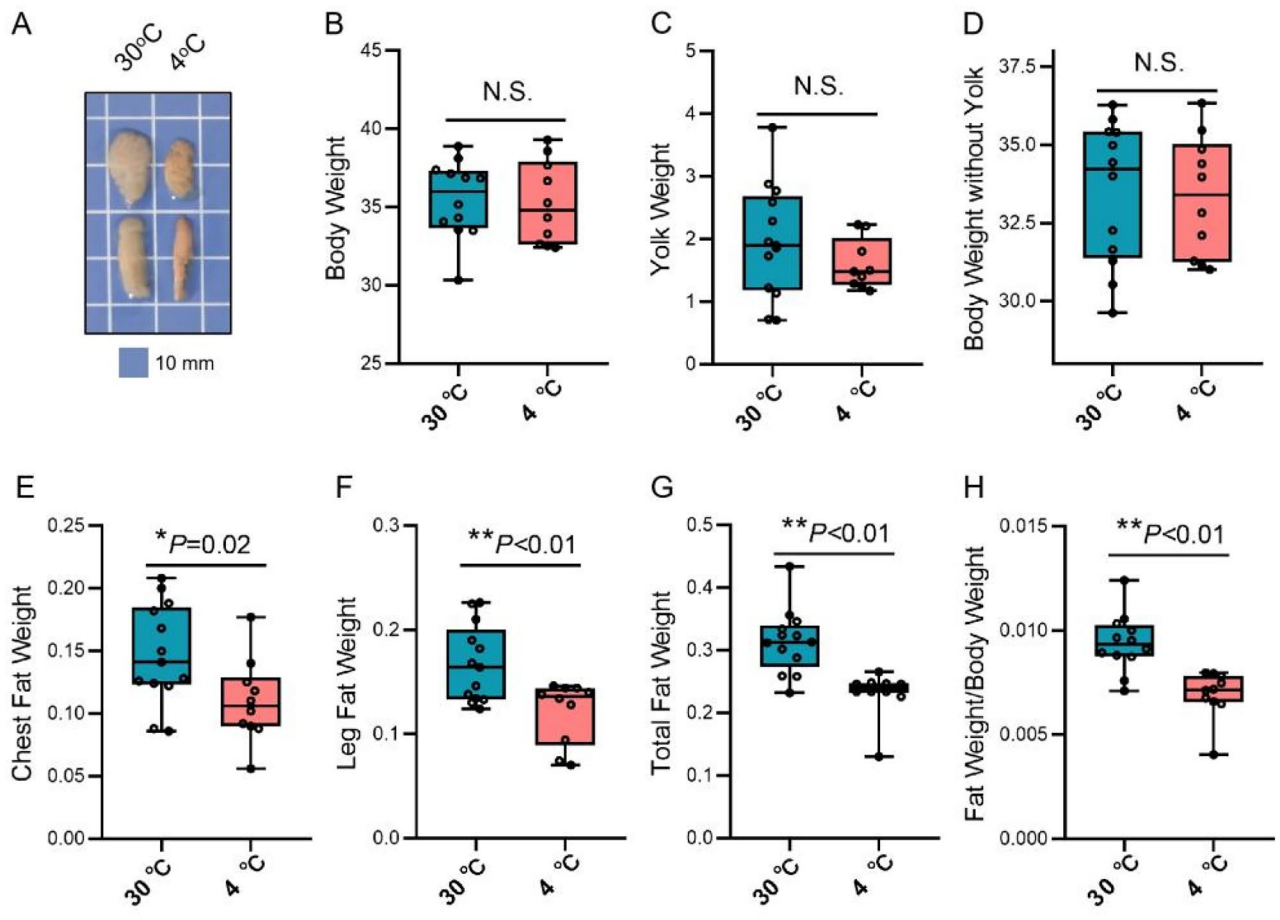
The effects of cold stress on chick subcutaneous adipose tissue. (A) The size of chick subcutaneous adipose tissue at a temperature of 30 or 4 °C; (B–H) the body weight, yolk weight, body weight without yolk, chest fat weight, leg fat weight, total subcutaneous adipose tissue weight and relative subcutaneous adipose tissue weight.

Previous studies have shown that the interaction between adipocytes and macrophages in subcutaneous adipose tissue of chicks is involved in fatty acid transport, but whether the interaction between adipocytes and macrophages is involved in other biological processes in subcutaneous adipose tissue of chicks. In this study, a cold stress model was established to evaluate the physiological function of subcutaneous adipose tissue of chicks by measuring the weight of tissues and organs associated with lipid metabolism, and the results are shown in Fig. 1. After 36 h cold treatment, the weight of chest adipose tissue, leg adipose tissue, total adipose tissue and total adipose tissue/body weight were reduced (*P* < 0.05). The body weight, yolk weight and body weight without yolk were no significant difference between 30 °C group and 4 °C group. The results indicated that subcutaneous adipose tissue of chicks was relatively active in response to environmental cold stress.

### Cold stress induced immune infiltration of subcutaneous adipose tissue in chicks

**Figure 2.**
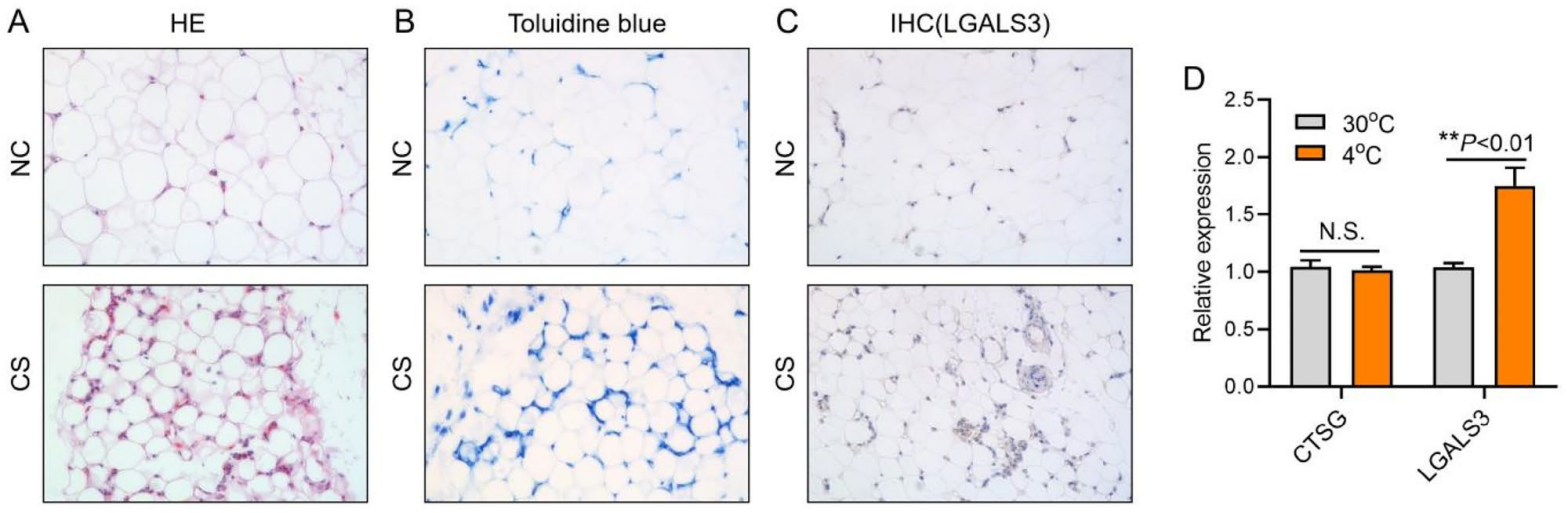
The effects of cold stress on immune infiltration of chick subcutaneous adipose tissue. (A) HE staining of chick subcutaneous adipose tissue at 30 and 4 °C; (B) toluidine blue staining of chick subcutaneous adipose tissue at 30 and 4 °C; (C) LGALS3 immunohistochemical staining of chick subcutaneous adipose tissue at 30 and 4 °C and (D) relative expression levels of chick subcutaneous adipose tissue *CTSG* and *LGALS3* genes at 30 and 4 °C.

In order to confirm whether macrophages participate in lipid metabolism homeostasis under cold stress, the involvement of mast cells and macrophages in adipose tissue was evaluated by HE staining, toluidine blue staining and LGALS3 immunohistochemical staining. HE staining showed that the subcutaneous adipose tissue of chicks exhibited a phenotype similar to the Browning of white adipose in rodents under cold stress (Fig. 2A). The results of toluidine blue staining showed that mast cells were not the main type of immune infiltration in adipose tissue under cold stress (Fig. 2B). The results of LGALS3 immunohistochemical staining showed that a large number of macrophages were recruited into the subcutaneous adipose tissue of chicks, suggesting that macrophages may participate in lipid homeostasis under cold stress (Fig. 2C). Mast cell marker gene *CTSG* and chicken macrophage marker gene *LGALS3* were detected in chicken subcutaneous adipose tissue by RT-qPCR, and the results were consistent with toluidine blue staining and LGALS3 immunohistochemical staining, which were indicated that macrophages are the main form of immune infiltration of adipose tissue under chicken skin of environmental cold stress (Fig. 2D).

### Cold stress induced infrared thermal imaging of subcutaneous adipose tissue in chicks

**Figure 3.**
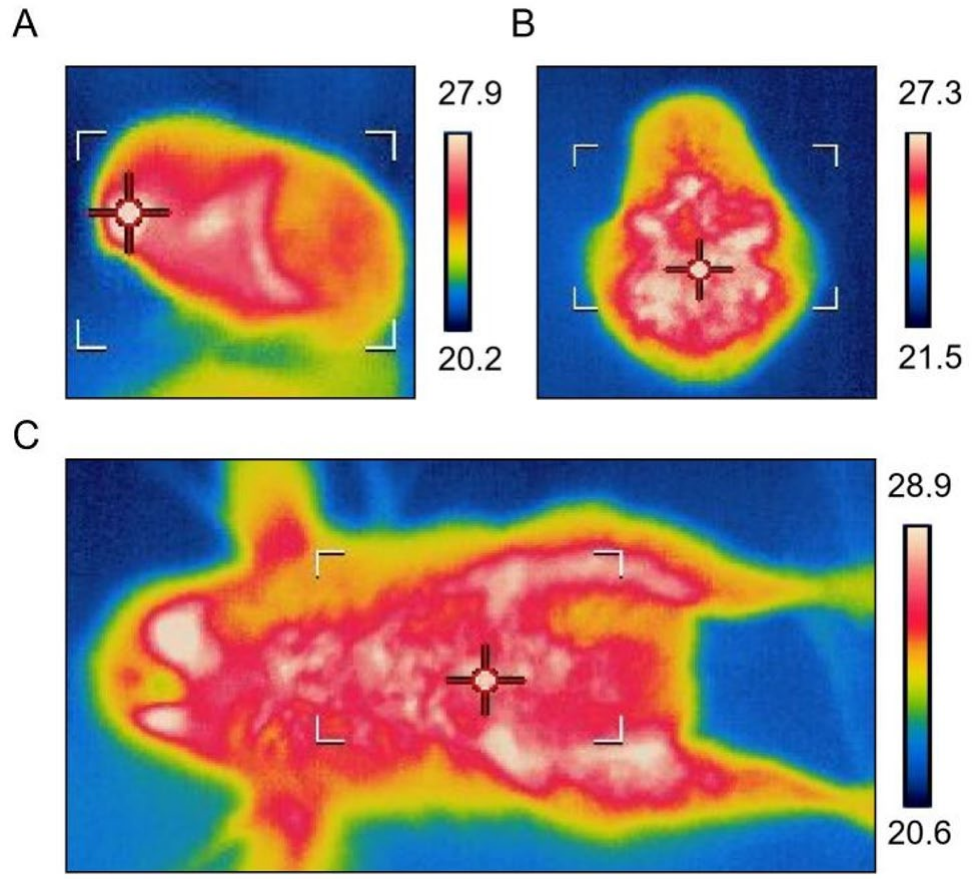
Thermal imaging of chick subcutaneous adipose tissue.

In order to evaluate whether the subcutaneous adipose tissue of chicks has additional thermogenic functions similar to those designed for browning of ruminant white fat, thermal radiation images were obtained on the left, dorsal and ventral sides of chicks by infrared thermal imager. The results showed that the thermal radiation levels in the major subcutaneous fat accumulation areas (neck, abdomen and legs) were higher in the chicks (Fig. 3). It is speculated that the subcutaneous adipose tissue of chicks may participate in the function of body heat production.

### Cold stress induced lipid metabolism of subcutaneous adipose tissue in chicks

**Figure 4.**
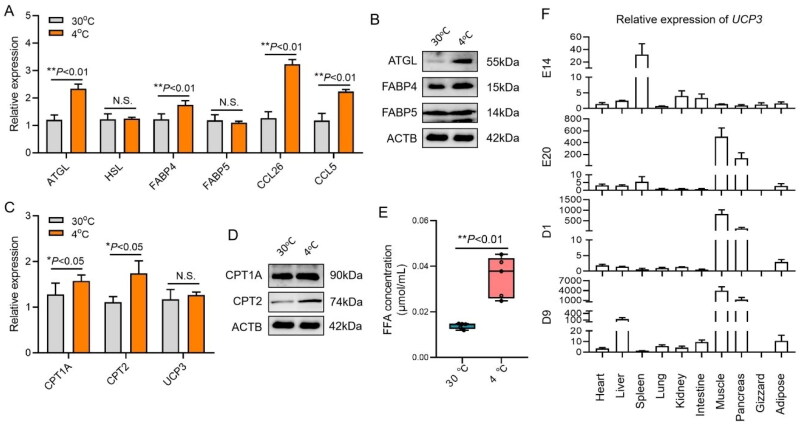
The effects of cold stress on fatty acid metabolism of chick subcutaneous adipose tissue. (A,B) Lipidolysis and chemokines secretion of chick subcutaneous adipose tissue at 30 and 4 °C; (C,D) fatty acid β-oxidation and themogenesis of chick subcutaneous adipose tissue at 30 and 4 °C; (E) free fatty acid concentration of chick serum at 30 and 4 °C and (F) the expression of UCP3 between four development stages in 10 tissues.

The mRNA expressions of *ATGL* and *FABP4* in chick subcutaneous adipose tissue were significantly upregulated of environmental cold stress, indicating that the decomposition level of triglyceride (TG) was significantly increased (*P* < 0.05) (Fig. 4A). At the same time, the expression levels of *CCL26* and *CCL5* were also significantly upregulated (*P* < 0.05). The upregulation of *CCL26* may be attributed to the release of *CCL26* by adipocytes due to lipidolysis under cold stress to recruit a large number of macrophages, and the increase in the expression level of *CCL26* in tissues caused by the large recruitment of macrophages may also exist. The protein expressions of ATGL in chick subcutaneous adipose tissue were significantly upregulated of environmental cold stress (Fig. 4B). As a marker gene of macrophages, CCL5 is only expressed in macrophages, and the expression level is low in adipocytes, while CCL26 is expressed in both adipocytes and macrophages of chickens. In the cold stress environment, the expression of *CCL5* was significantly upregulated, which proved that a large number of macrophages were recruited into the adipose tissue, resulting in an increase in the expression level of CCL5 (Fig. 4A,B).

The function of macrophage recruitment in adipose tissue of environmental cold stress could not be limited to the process of lipid transport. By evaluating the expression levels of *CPT1A*, *CPT2* and *UCP3* genes in chick subcutaneous adipose tissue, it was found that the expression levels of *CPT1A* and *CPT2* genes were significantly upregulated (*P* < 0.05). The results showed that the β oxidation of fatty acid in chick subcutaneous adipose tissue was significantly increased of environmental cold stress, but the expression level of *UCP3* was not significantly changed (*P* > 0.05) (Fig. 4C,D). The FFA concentration of chick serum was upregulated of environmental cold stress (Fig. 4E). As the major type of UCP family, *UCP3* was upregulated after hatching (Fig. 4F).

### Expression of thermogenic gene in adipocyte and macrophage coculture mode

**Figure 5.**
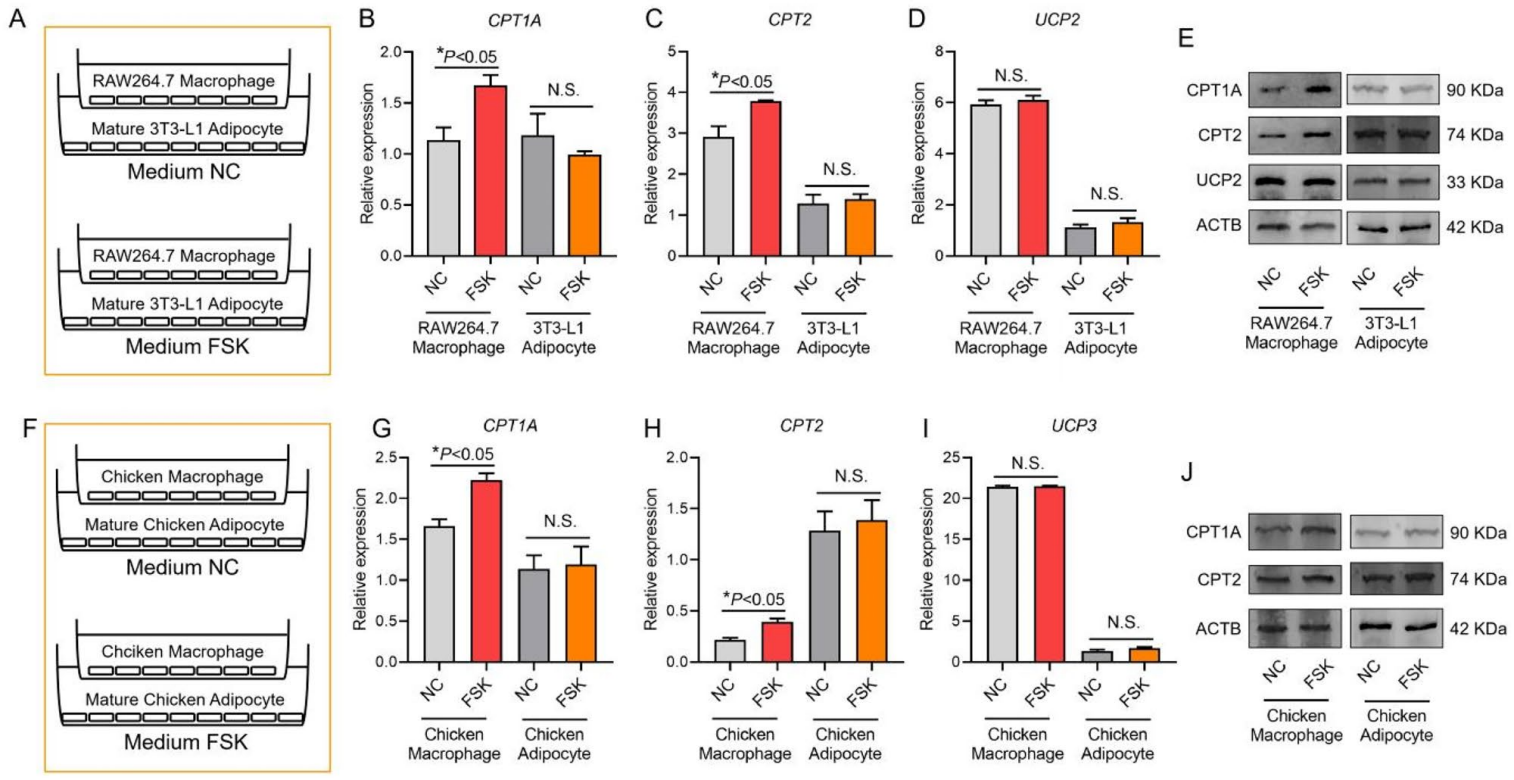
Fatty acid metabolism in coculture of mature adipocytes and macrophages. (A–E) Fatty acid metabolism in coculture of mature 3T3-L1 adipocytes and RAW264.7 macrophages and (F–J) fatty acid metabolism in coculture of mature chicken adipocytes and chicken macrophages.

In order to evaluate whether the interaction between adipocytes and macrophages is directly involved in the thermogenesis of subcutaneous adipose tissue of chicks, the expression level of genes related to fatty acid metabolism and thermogenesis was detected by using the interaction cell model of mature adipocytes and macrophages. *CPT1A*, *CPT2* and *UCP2* gene expression levels were detected by the coculture system of mature 3T3-L1 adipocytes and RAW264.7 macrophages. The results showed that fatty acid metabolism level of RAW264.7 macrophages was significantly improved in the coculture system. However, UCP2-mediated mitochondrial uncoupling level was not significantly increased (*P* > 0.05), suggesting that macrophages did not generate a lot of heat through uncoupling effect while fatty acid metabolism level was significantly increased (Fig. 5A–E). *CPT1A*, *CPT2* and *UCP3* gene expression levels were detected by the coculture system of mature chicken adipocytes and chicken macrophages. The results of mature chicken adipocytes and chicken macrophages coculture system were consistent with the results of mature 3T3-L1 adipocytes and RAW264.7 macrophages coculture system, the fatty acid metabolism level of chicken macrophages was significantly improved. However, UCP3-mediated mitochondrial uncoupling was not significantly increased (*P* > 0.05), suggesting that macrophages did not generate a lot of heat through the uncoupling effect while the level of fatty acid metabolism was significantly increased. There were no significant changes in *CPT1A*, *CPT2* and *UCP3* gene expression levels in chicken adipocytes in the coculture system (*P* > 0.05), indicating that the presence of macrophages did not improve the fatty acid metabolism and mitochondrial uncoupling level of chicken adipocytes (Fig. 5F–J).

### Cold stress induced subcutaneous adipose tissue fibrosis and angiogenesis in chicks

**Figure 6.**
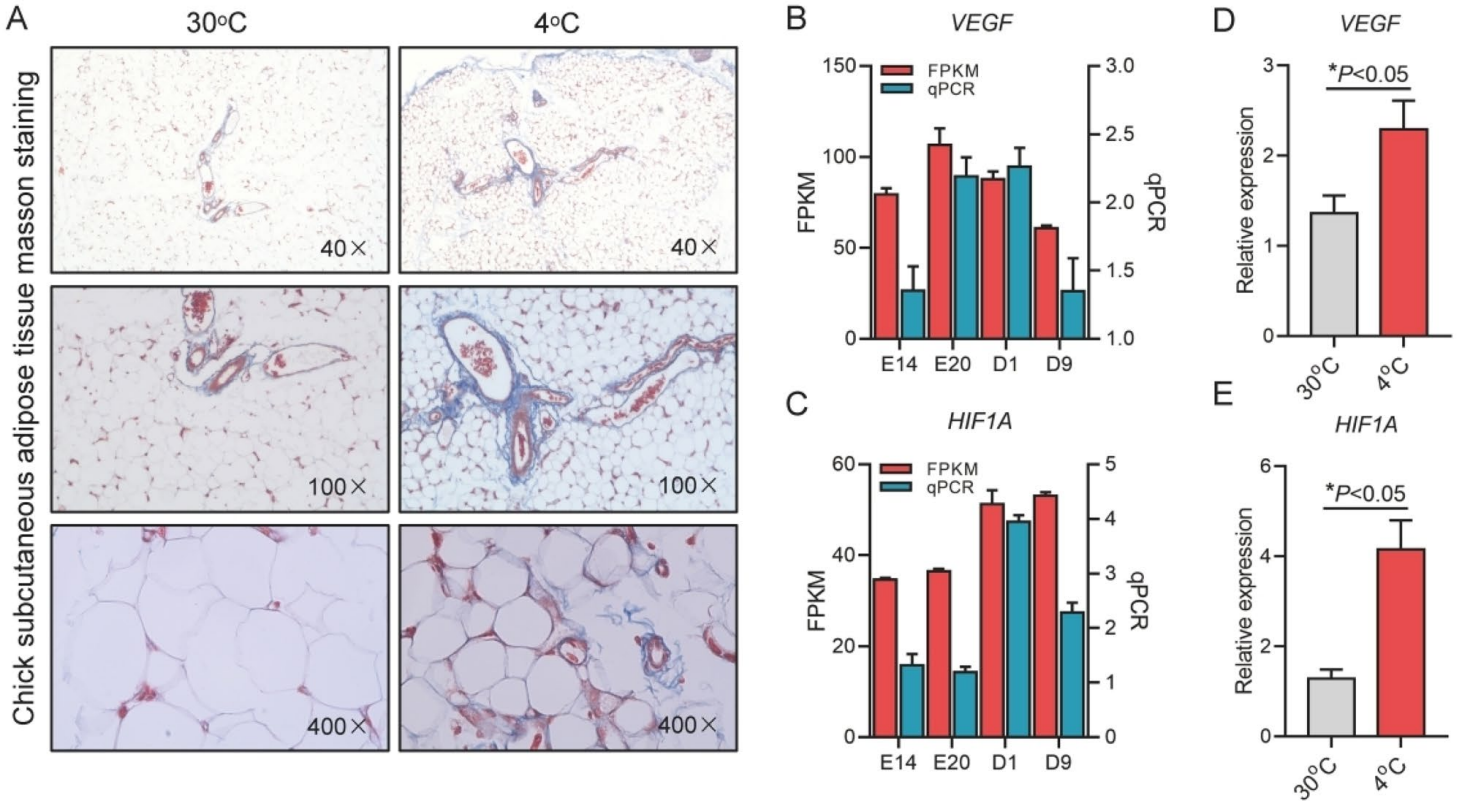
The effects of cold stress on fibrosis and stimulate angiogenesis. (A) Masson staining of chick subcutaneous adipose tissue at 30 and 4 °C; (B,C) qPCR of *HIF1A* and *VEGF* expression chick subcutaneous adipose tissue of chicken embryos and chicks at four different developmental stages; (D,E) The expression of *HIF1A* and *VEGF* in chick subcutaneous adipose tissue at 30 and 4 °C.

To evaluate the role of adipocyte and macrophage interaction in the process of subcutaneous adipose tissue fibrosis and angiogenesis in chicks, Masson staining was used to evaluate the subcutaneous adipose tissue fibrosis level of chicks of environmental cold stress, and the results showed that the subcutaneous adipose tissue fibrosis level of chicks of environmental cold stress was significantly increased, which could be related to the mass recruitment of macrophages in adipose tissue of chicks (Fig. 6A). The expression levels of *HIF1A* and *VEGF* genes in chick subcutaneous adipose tissue of chicken embryos and chicks at four different developmental stages were detected by RT-qPCR, the results showed that the expression levels of *HIF1A* and *VEGF* genes showed an upregulated trend before and after the birth of chicks (Fig. 6B,C). In addition, the expression levels of *HIF1A* and *VEGF* genes in subcutaneous adipose tissue of environmental cold stress were detected, and the results showed that cold stress significantly increased the expression levels of *HIF1A* and *VEGF* genes in subcutaneous adipose tissue of chicks (*P* < 0.05). It was shown that angiogenesis mediated by *HIF1A–VEGF* pathway was significantly enhanced in chicks under cold stress (Fig. 6D,E).

### Expression of HIF1A and VEGF in adipocyte and macrophage coculture

In order to evaluate the role of adipocyte and macrophage interaction in adipose tissue angiogenesis, the expression levels of *HIF1A* and *VEGF* genes were detected using the mature adipocyte and macrophage interaction cell model.

**Figure 7.**
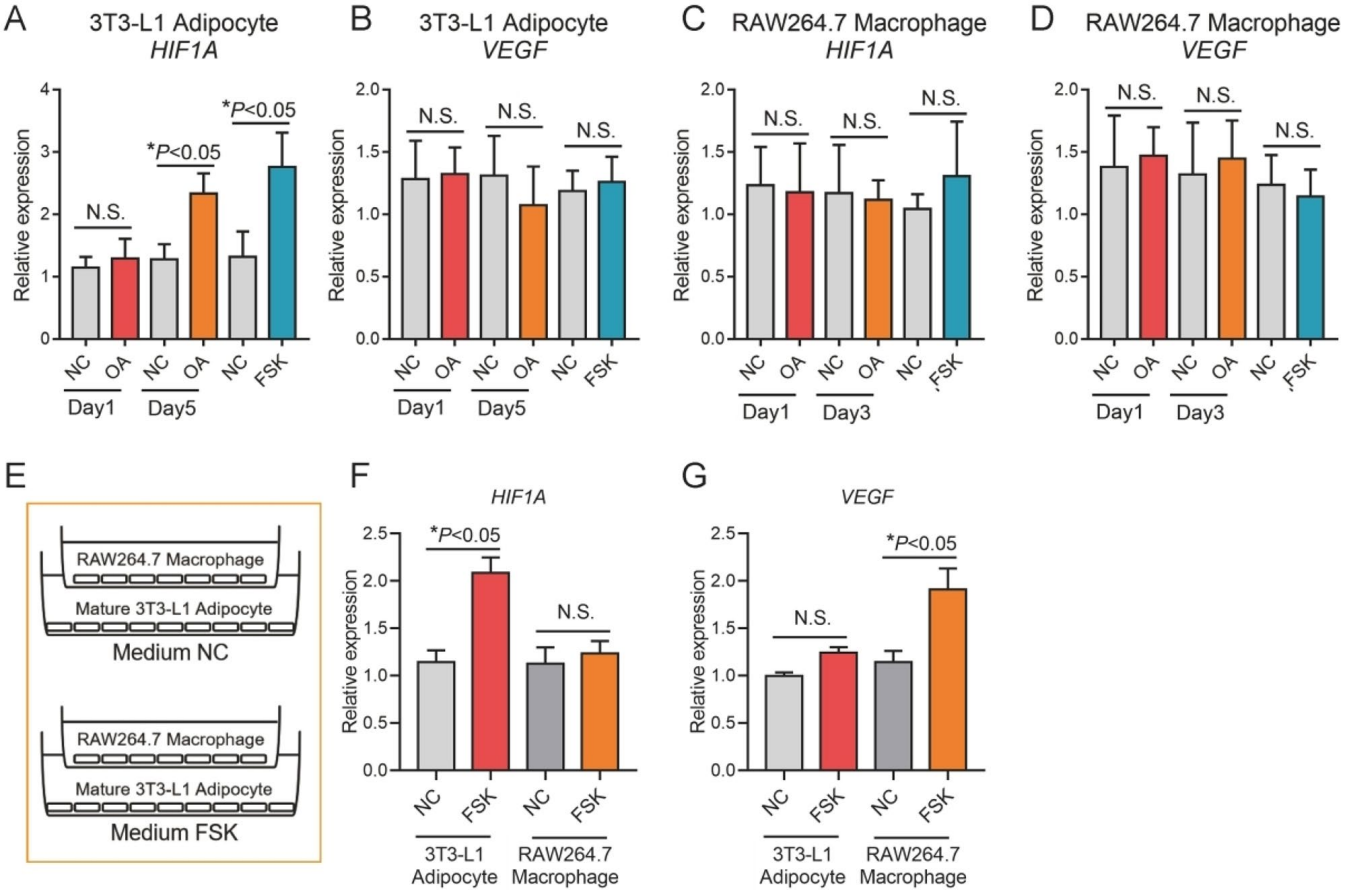
3T3-L1 adipocytes and RAW264.7 macrophages participate in angiogenesis. (A,B) The expression of HIF1A and VEGF in 3T3-L1 adipocyte treated with OA and FSK; (C,D) the expression of HIF1A and VEGF in RAW264.7 macrophages treated with OA and FSK and (E–G) the expression of HIF1A and VEGF in coculture of 3T3-L1 adipocyte and RAW264.7 macrophages treated with OA and FSK.

Firstly, the expression of *HIF1A* and *VEGF* genes was evaluated by short-term and long-term stimulation of fatty acid in 3T3-L1 adipocytes and RAW264.7 macrophages. The results showed that the expression level of *HIF1A* gene in 3T3-L1 adipocytes was significantly increased after long-term treatment by FFA (*P* < 0.05). The increased expression level may be related to the increase of fat cell size. The expression level of *HIF1A* gene was significantly increased after induction of lipidolysis by FSK in mature 3T3-L1 adipocytes (*P* < 0.05), indicating that lipidolysis is also an inducer of *HIF1A* gene (Fig. 7A–D). The changes of *HIF1A* and *VEGF* gene expression levels in mature 3T3-L1 adipocytes and RAW264.7 macrophages were detected in the coculture system, and significantly increased *HIF1A* gene expression levels were detected in 3T3-L1 adipocytes in the coexpression system (*P* < 0.05). The increase of *HIF1A* gene expression in RAW264.7 macrophages was not detected. In the coexpression system, increased *VEGF* gene expression was detected in RAW264.7 macrophages, but not in 3T3-L1 adipocytes (Fig. 7E-G).

**Figure 8.**
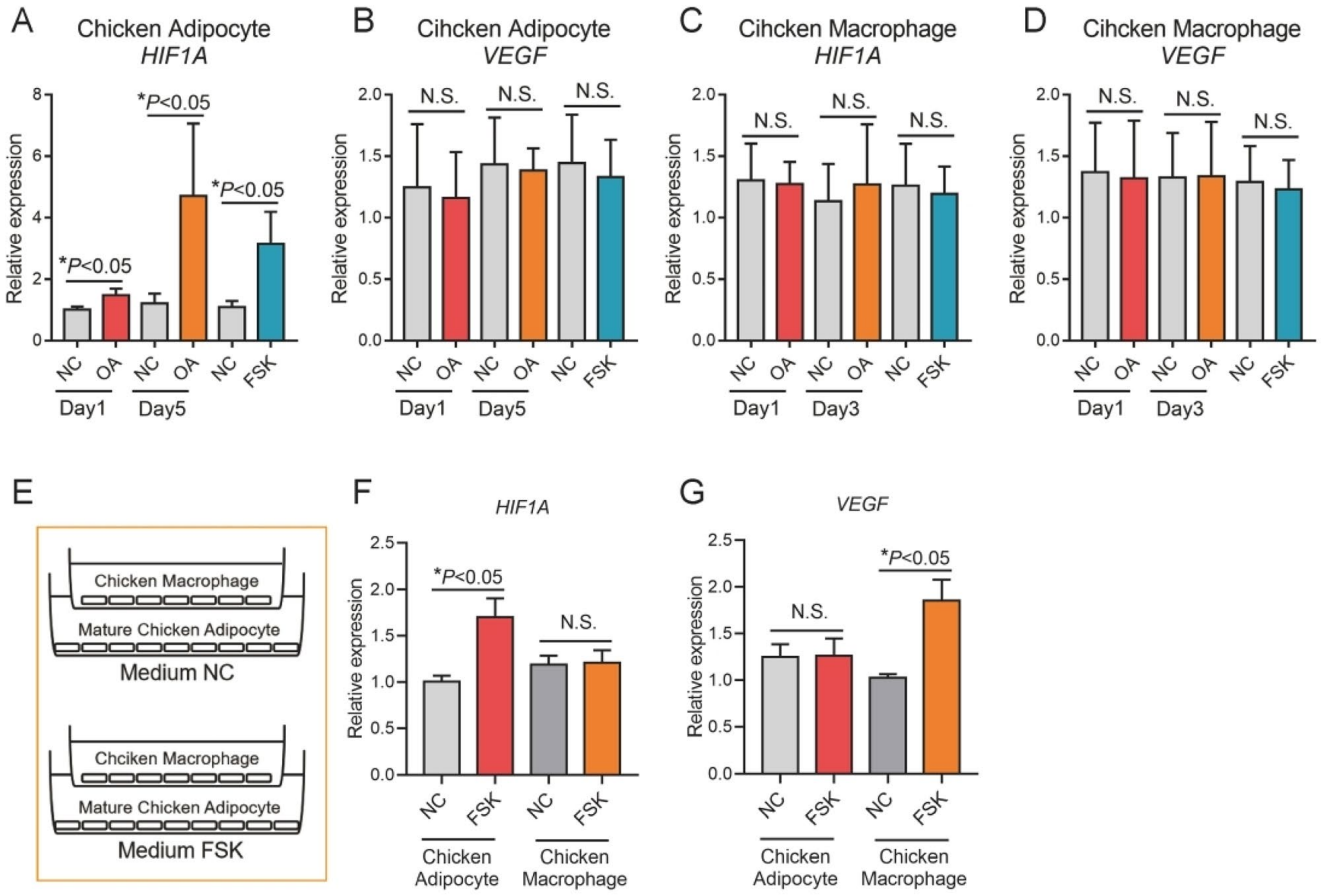
Chicken adipocytes and macrophages participate in angiogenesis. (A,B) The expression of HIF1A and VEGF in chicken adipocyte treated with OA and FSK; (C,D) the expression of HIF1A and VEGF in chicken macrophages treated with OA and FSK and (E–G) the expression of HIF1A and VEGF in coculture of chicken adipocyte and macrophages treated with OA and FSK.

Secondly, the expression of *HIF1A* and *VEGF* genes was evaluated by short-term and long-term stimulation of chicken adipocytes and chicken macrophages with fatty acids. The results showed that the expression level of *HIF1A* gene was significantly increased after long-term treatment of chicken adipocytes with FFA (*P* < 0.05), and the increase of expression level may also be related to the increase of adipocyte volume. The expression level of *HIF1A* gene was significantly increased after adipocyte lipidolysis was induced by FSK (*P* < 0.05), indicating that lipidolysis is also a inducer of *HIF1A* gene in adipocytes of chicken (Fig. 8A–D). The changes of *HIF1A* and *VEGF* gene expression levels were detected by the coculture system of mature chicken adipocytes and chicken macrophages. The *HIF1A* gene expression level was significantly increased in chicken adipocytes in the coexpression system (*P* < 0.05), but the increase of *HIF1A* gene expression level in chicken macrophages was not detected. Increased *VEGF* gene expression was detected in chicken macrophages in the coexpression system, but not in chicken adipose cells (Fig. 8E-G).

## Discussion

The three elements of adaptive thermogenesis in adipose tissue are catecholamine synthesis, TG decomposition and UCPs activation. Chicken adipose tissue is mainly divided into subcutaneous adipose tissue and abdominal adipose tissue, both of which are lipid deposition organs with NST material basis. Sympathetic nerve distribution exists in adipose tissue, which has the basis of NST regulation. However, the expression integrity of catecholamine metabolic enzyme system in chicken adipose tissue is controversial. The expression cell lineage of catecholamine metabolic enzyme system in chicken adipose tissue and the regulation type of catecholamine ligand-receptor have not been reported. At present, the existence of brown adipose tissue in chickens is still controversial, but the deletion of chicken *UCP1* gene is an important obstacle to the NST function of chicken adipose tissue.^12^ Only *UCP3* gene has been found in chickens, and most studies have focused on muscle tissue, and only a small amount of *UCP3* gene is expressed in adipose tissue, and whether it can be activated by cold stress signal has not been reported.

At present, the understanding of adipose tissue adaptive thermogenesis function is still in the initial stage, and the study of adipose tissue adaptive thermogenesis function based on the cell heterogeneity of adipose tissue is of great significance for further understanding of adipose tissue function. The adaptive thermogenic function of adipose tissue can be divided into two forms: ST and NST. There are special thermogenic adiopocyte groups in adipose tissue, which can enhance the body’s adaptive thermogenic level through mitochondrial uncoupling mediated by UCP1^13^; Adaptive thermogenesis mediated by the interaction between other cell types in adipose tissue and adipocyte.^14^ Studies have shown that catecholamines are the main inducer of adipose tissue thermogenesis, but the source of catecholamines in adipose tissue is still controversial. Some studies have found that the main source of catecholamines in adipose tissue may be macrophages^15^^,^^16^; however, the integrity of expression of a series of catecholamine metabolic enzymes in macrophages is also controversial, and some studies have proposed that catecholamine metabolic enzymes in adipose tissue are coregulated by adipocytes and macrophages.^3^

Rodents generate a large amount of heat through the browning process of white fat to cope with temperature changes in the external environment.^17^ Through the establishment of chicken cold stress model, the results showed that the fatty acid metabolism level of chicken subcutaneous adipose tissue increased significantly in cold environment, but the expression level of UCP did not change significantly. Chicken subcutaneous adipose tissue recruited a large number of macrophages, it was inferred that the interaction between adipocytes and macrophages may be related to adaptive thermogenesis of chicken adipose tissue. By analysing the expression levels of fatty acid metabolic enzymes and UCPs in the interaction between adipocytes and macrophages, it was found that the ability of fatty acid metabolism of macrophages in lipid decomposition microenvironment increased significantly (*P* < 0.05). However, the level of mitochondrial uncoupling heat production did not increase, indicating that the interaction between adipocytes and macrophages is not the direct cause of adipose tissue browning.

Environment temperature is an important inducement of angiogenesis in tissues and organs, and the improvement of vascularization level can significantly enhance the transport level of energy substances.^18^^,^^19^ Through the interaction experiment between adipocytes and macrophages, this study revealed that although macrophages did not directly participate in the thermogenic effect of adipose tissue, they could enhance the transport capacity of energy substances in adipose tissue by enhancing angiogenesis.^20^ The angiogenesis mediated by HIF1A–VEGF was analysed by the interaction model between adipocytes and macrophages.^21^^,^^22^ The results showed that HIF1A in adipocytes could indirectly regulate the expression of VEGF in macrophages through some factors. this regulation mode goes beyond the regulation of adipose tissue angiogenesis at the present stage, but the molecular mechanism is not clear. It has been reported that FABP4 can act on the direct regulatory factors of VEGF and participate in the process of macrophage-mediated angiogenesis, but there is no complete evidence at the level of cell interaction.^23^ Macrophages can be polarized into different types involved in tissue remodelling due to their strong plasticity, It has been confirmed that it is involved in lipid deposition, lipid transport, cellular inflammation, etc.^24^^,^^25^ The molecular mechanism of angiogenesis mediated by HIF1A–VEGF signal pathway in adipocytes and macrophages remains to be further studied.

In conclusion, this study constructed the model of chick in cold condition to explore the physiological significance of the massive deposition of subcutaneous adipose tissue during the chick stage. According to the previous research, the reason for the large deposition of subcutaneous adipose tissue in chicks may be the rapid absorption of chick yolk after the birth of chicks. So it is inferred that the deposition of subcutaneous adipose tissue in chicks may be the energy storage tissue to cope with the postnatal environment. Current studies on avian adipose tissue have focused on abdominal fat deposition and rarely involved subcutaneous adipose tissue-related functions, especially to evaluate whether chick adipose tissue has thermogenic-related functions.^26^^,^^27^ In this study, the thermogenic-related function of subcutaneous adipose tissue in chicks by means related to histology and cell biology, the histological results indicated that, the subcutaneous adipose tissue of chicks is rapidly consumed under cold stress conditions; The results of immune infiltration indicated that, a large number of macrophages were recruited in the adipose tissue of chicks under cold stress conditions; The results of the tissue level and the cell interaction level indicate that, interaction between adipocytes and macrophages is only involved in mitochondrial fatty acid beta oxidation in adipose tissue, was not involved in the fatty acid uncoupling thermal production effect. At the same time, the interaction between adipocytes and macrophages may participate in the fibrosis and angiogenesis of the subcutaneous adipose tissue in chicks via HIF1A–VEGF. From this result, chick adipose tissue may enhance the vascularization level of adipose tissue through the interaction between adipocytes and macrophages. This effect may benefit the tissue transport of fatty acids after adipose tissue lipid decomposition and also suggests that chick adipose tissue may not have the relevant thermogenesis function in dealing with cold stress. In the future, we may study the signalling pathways related to lipid deposition and decomposition in subcutaneous adipose tissue to explore the molecular mechanism of efficient lipid deposition and decomposition in chicks, so as to provide new ideas for the study of lipid deposition.

## Conclusion

Under cold stress condition, macrophage infiltration level in chick subcutaneous adipose tissue was significantly increased (*P* < 0.05), the interaction between adipocyte and macrophage could enhanced angiogenesis by HIF1A–VEGF signalling pathway, which did not directly participate in fatty acid metabolism and thermogenesis in adipose tissue.

## Supplementary Material

supplementary-file--Table-S1.docx

## Data Availability

The original contributions presented in the study are included in the article; further inquiries can be directed to the corresponding author.

## References

[1] Zhao H, Wu M, Tang X, et al. Function of chick subcutaneous adipose tissue during the embryonic and posthatch period. *Front Physiol*. 2021;12:684426. 34239450 10.3389/fphys.2021.684426 PMC8258255

[2] Chait A, den Hartigh LJ. Adipose tissue distribution, inflammation and its metabolic consequences, including diabetes and cardiovascular disease. *Front Cardiovasc Med*. 2020;7:22. 32158768 10.3389/fcvm.2020.00022 PMC7052117

[3] Gomes A, Leite F, Ribeiro L. Adipocytes and macrophages secretomes coregulate catecholamine synthesizing enzymes. *Int J Med Sci*. 2021;18(3):582–592. 33437193 10.7150/ijms.52219 PMC7797554

[4] Habibu B, Aliyu A, Idris SY, et al. Thermoregulation in periparturient rabbit does and their neonatal kits with different litter sizes during West African winter. *Anim Biotechnol*. 2023;34(9):4357–4366. 36459437 10.1080/10495398.2022.2150200

[5] Zhao H, Wu M, Tang X, et al. RNA-seq based transcriptome analysis reveals the cross-talk of macrophage and adipocyte of chicken subcutaneous adipose tissue during the embryonic and post-hatch period. *Front Immunol*. 2022;13:889439. 35911745 10.3389/fimmu.2022.889439 PMC9334849

[6] Chouchani ET, Kajimura S. Metabolic adaptation and maladaptation in adipose tissue. *Nat Metab*. 2019;1(2):189–200. 31903450 10.1038/s42255-018-0021-8 PMC6941795

[7] Cairó M, Villarroya J. The role of autophagy in brown and beige adipose tissue plasticity. *J Physiol Biochem*. 2020;76(2):213–226. 31811543 10.1007/s13105-019-00708-1

[8] Bartelt A, Heeren J. Adipose tissue browning and metabolic health. *Nat Rev Endocrinol*. 2014;10(1):24–36. 24146030 10.1038/nrendo.2013.204

[9] Ouchi Y, Chowdhury VS, Cockrem JF, Bungo T. Av-UCP single nucleotide polymorphism affects heat production during cold exposure in chicks. *J Therm Biol*. 2021;98:102909. 34016336 10.1016/j.jtherbio.2021.102909

[10] Sotome R, Hirasawa A, Kikusato M, et al. In vivo emergence of beige-like fat in chickens as physiological adaptation to cold environments. *Amino Acids*. 2021;53(3):381–393. 33598768 10.1007/s00726-021-02953-5 PMC7979618

[11] Joubert R, Métayer-Coustard S, Crochet S, et al. Regulation of the expression of the avian uncoupling protein 3 by isoproterenol and fatty acids in chick myoblasts: possible involvement of AMPK and PPARalpha? *Am J Physiol Regul Integr Comp Physiol*. 2011;301(1):R201–R208. 21508290 10.1152/ajpregu.00087.2010

[12] Mezentseva NV, Kumaratilake JS, Newman SA. The brown adipocyte differentiation pathway in birds: an evolutionary road not taken. *BMC Biol*. 2008;6(1):17. PMID: 18426587; PMCID: PMC2375860. 18426587 10.1186/1741-7007-6-17 PMC2375860

[13] Sun W, Dong H, Balaz M, et al. snRNA-seq reveals a subpopulation of adipocytes that regulates thermogenesis. *Nature*. 2020;587(7832):98–102. 33116305 10.1038/s41586-020-2856-x

[14] Cho YK, Son Y, Kim SN, et al. MicroRNA-10a-5p regulates macrophage polarization and promotes therapeutic adipose tissue remodeling. *Mol Metab*. 2019;29:86–98. 31668395 10.1016/j.molmet.2019.08.015 PMC6734158

[15] Nguyen KD, Qiu Y, Cui X, et al. Alternatively activated macrophages produce catecholamines to sustain adaptive thermogenesis. *Nature*. 2011;480(7375):104–108. 22101429 10.1038/nature10653 PMC3371761

[16] Reitman ML. How does fat transition from white to beige? *Cell Metab*. 2017;26(1):14–16. 28683281 10.1016/j.cmet.2017.06.011 PMC6088243

[17] Gambaro SE, Zubiría MG, Giordano AP, et al. Role of spexin in white adipose tissue thermogenesis under basal and cold-stimulated conditions. *Int J Mol Sci*. 2024;25(3):1767. 38339044 10.3390/ijms25031767 PMC10855774

[18] Shamsi F, Zheng R, Ho LL, Chen K, Tseng YH. Comprehensive analysis of intercellular communication in the thermogenic adipose niche. *Commun Biol*. 2023;6(1):761. 37479789 10.1038/s42003-023-05140-2 PMC10361964

[19] Wang C, Wu Y, Li Y, et al. Smad4-mediated angiogenesis facilitates the beiging of white adipose tissue in mice. *iScience*. 2023;26(3):106272. 36915676 10.1016/j.isci.2023.106272 PMC10005906

[20] Martin P, Gurevich DB. Macrophage regulation of angiogenesis in health and disease. *Semin Cell Dev Biol*. 2021;119:101–110. 34330619 10.1016/j.semcdb.2021.06.010

[21] Ahmadi-Kani Golzar F, Fathi R, Mahjoub S. High-fat diet leads to adiposity and adipose tissue inflammation: the effect of whey protein supplementation and aerobic exercise training. *Appl Physiol Nutr Metab*. 2019;44(3):255–262. 30107135 10.1139/apnm-2018-0307

[22] Wang X, de Carvalho Ribeiro M, Iracheta-Vellve A, et al. Macrophage-specific hypoxia-inducible factor-1α contributes to impaired autophagic flux in nonalcoholic steatohepatitis. *Hepatology*. 2019;69(2):545–563. 30102772 10.1002/hep.30215 PMC6351177

[23] Elmasri H, Karaaslan C, Teper Y, et al. Fatty acid binding protein 4 is a target of VEGF and a regulator of cell proliferation in endothelial cells. *FASEB J*. 2009;23(11):3865–3873. 19625659 10.1096/fj.09-134882 PMC2775007

[24] Wang B, Tang X, Yao L, et al. Disruption of USP9X in macrophages promotes foam cell formation and atherosclerosis. *J Clin Invest*. 2022;132(10):e154217. 35389885 10.1172/JCI154217 PMC9106359

[25] Westerterp M, Tall AR. A new pathway of macrophage cholesterol efflux. *Proc Natl Acad Sci U S A*. 2020;117(22):11853–11855. 32424095 10.1073/pnas.2007836117 PMC7275720

[26] Chi Y, Xu Y, Luo F, Lin Y, Li Z. Molecular cloning, expression profiles and associations of KLF6 gene with intramuscular fat in Tibetan chicken. *Anim Biotechnol*. 2020;31(1):67–75. 30501383 10.1080/10495398.2018.1540428

[27] Rahnama M, Bouyeh M, Kadim I, et al. Effect of dietary inclusion of lecithin with choline on physiological stress of serum cholesterol fractions and enzymes, abdominal fat, growth performance, and mortality parameters of broiler chickens. *Anim Biotechnol*. 2020;31(6):483–490. 31230524 10.1080/10495398.2019.1622557

[28] Amorim NRT, Souza-Almeida G, Luna-Gomes T, et al. Leptin elicits in vivo eosinophil migration and activation: key role of mast cell-derived PGD2. *Front Endocrinol*. 2020;11:572113. 10.3389/fendo.2020.572113 PMC7551309 33117286

