# Supplementary material for: Interaction between adipocytes and macrophages participates in chick subcutaneous adipose tissue angiogenesis under cold stress conditions
Source: Anim Biotechnol. 2024 Sep 17;35(1):2400212. doi: 10.1080/10495398.2024.2400212 (PMC12674257; doi:10.1080/10495398.2024.2400212)
Supplement: supplementary-file--Table-S1.docx [file LABT_A_2400212_SM7520.docx]

**Table S1** Primers used in this study

| **Genes** | **Accession number** | **Sequencing(5'-3')** | **Notes** | **Species** |
| --- | --- | --- | --- | --- |
| gga-*CTSG* | [XM_423728.6](https://www.ncbi.nlm.nih.gov/nuccore/XM_423728.6) | F: ACACTGCTTGGAGGGAAACAT | RT-qPCR | Chicken |
|  |  | R: GAGCTTGACCTTCGCTGTCA |  |  |
| gga-*LGALS3* | [NM_001302800.2](https://www.ncbi.nlm.nih.gov/nuccore/NM_001302800.2) | F: CCCCGTTTCAAGGAAGACCA | RT-qPCR | Chicken |
|  |  | R: CAGAGCACCTGGAGCTTGAA |  |  |
| *gga-ATGL* | NM_001113291.1 | F: TGCGTGGAGTGAGATATGTTGA | RT-qPCR | Chicken |
|  |  | R: TTGCGAAGGTTGAATTGGAT |  |  |
| *gga-HSL* | XM_025155301.1 | F: GTCTCGGGTTCCAGTTCGTG | RT-qPCR | Chicken |
|  |  | R: CGTAGGACACCAACCCGATG |  |  |
| *gga-FABP4*  (*gga-A-FABP*) | NM_204290.1 | F: GCCAAGCCTAATTTAACTATCA | RT-qPCR | Chicken |
|  |  | R: CAGCAGGTTCCCATCCAC |  |  |
| gga-*FABP5* | [NM_204150.1](https://www.ncbi.nlm.nih.gov/nuccore/NM_204150.1) | F: AGCATGGCGAAACCAGATGT | RT-qPCR | Chicken |
|  |  | R: AGCTGACAAGGGTCTGAGTT |  |  |
| gga-*CCL26* | XM_415780.6 | F: ttcagatggcctacccacaac | RT-qPCR | Chicken |
|  |  | R: gactcctcggggtttacaca |  |  |
| gga-*CCL5* | NM_175827.2 | F: atgactgccgtagctgtgtc | RT-qPCR | Chicken |
|  |  | R: agcagcacacggttgtatca |  |  |
| *gga-CPT1A* | NM_001012898.1 | F: CTTGCCCTGCAGCTTGCT | RT-qPCR | Chicken |
|  |  | R: AGGCCTCGTATGTCAAAGAAATT |  |  |
| *gga-CPT2* | NM_001031287.2 | F: GCCTTCCCTCTTGGCTACCT | RT-qPCR | Chicken |
|  |  | R: TCTCAGCAATGCCCACGTATC |  |  |
| gga-*UCP3* | NM_001397652.1 | F: CGGGCCTCAGTTTCCCTAAG | RT-qPCR | Chicken |
|  |  | R: TAGAGATGACGCTTCGCTGC |  |  |
| mmu-*CPT1A* | NM_013495.2 | F: CGCCATACTGCTGTATCGTC | RT-qPCR | Mouse |
|  |  | R: ATGTGCCTGCTGTCCTTGA |  |  |
| mmu-*CPT2* | NM_009949.2 | F: GAGACTATTCGCCCAGCTTC | RT-qPCR | Mouse |
|  |  | R: GTATTTGGAGCACTCAGCCA |  |  |
| mmu-*UCP2* | NM_011671.5 | F: TCCCCTGTTGATGTGGTCAA | RT-qPCR | Mouse |
|  |  | R: CAGTGACCTGCGCTGTGGTA |  |  |
| gga-*HIF1A* | NM_001396327.1 | F: CGTCACCGACAAGAAGAGGATT | RT-qPCR | Chicken |
|  |  | R: CTTGTCAAGGTGGGCACTCA |  |  |
| gga-*VEGF* | NM_001110355.2 | F: GCAGAAGGTAAGGATGGGAGG | RT-qPCR | Chicken |
|  |  | R: GAATCCAGGCGAGGCTTCTG |  |  |
| mmu-*HIF1A* | NM_001313919.1 | F: CGCCTCTGGACTTGTCTCTT | RT-qPCR | Mouse |
|  |  | R: TCGACGTTCAGAACTCATCCT |  |  |
| mmu-*VEGF* | NM_001025250.3 | F: TATTCAGCGGACTCACCAGC | RT-qPCR | Mouse |
|  |  | R: AACCAACCTCCTCAAACCGT |  |  |
